# *Pseudomonas aestus* Isolation from the Nasal Cavity of a Cat with Chronic Rhinitis

**DOI:** 10.3390/vetsci11080382

**Published:** 2024-08-19

**Authors:** Raquel Abreu, Sofia Mouro, Joana F. Guerreiro, Sílvia A. Sousa, Jorge H. Leitão, Hugo Pissarra, Eva Cunha, Luís Tavares, Manuela Oliveira

**Affiliations:** 1 CIISA—Centre for Interdisciplinary Research in Animal Health, Faculty of Veterinary Medicine, University of Lisbon, 1300-477 Lisbon, Portugalmoliveira@fmv.ulisboa.pt (M.O.); 2 AL4AnimalS—Associate Laboratory for Animal and Veterinary Sciences, 1300-477 Lisbon, Portugal; 3 Veterinary Teaching Hospital, Faculty of Veterinary Medicine, University of Lisbon, 1300-477 Lisbon, Portugal; 4 Department of Bioengineering, IBB—Institute for Bioengineering and Biosciences, Instituto Superior Técnico, University of Lisbon, 1049-001 Lisbon, Portugal; 5 Associate Laboratory, i4HB—Institute for Health and Bioeconomy, Instituto Superior Técnico, University of Lisbon, 1049-001 Lisbon, Portugal; 6 cE3c—Centre for Ecology, Evolution and Environmental Changes & CHANGE—Global Change and Sustainability Institute, Faculty of Sciences, University of Lisbon, 1749-016 Lisbon, Portugal

**Keywords:** lymphoplasmacytic rhinitis, antimicrobial resistance, *Pseudomonas aestus*, *Pseudomonas aeruginosa*

## Abstract

**Simple Summary:**

A 9-years-old cat presenting signs of chronic respiratory disease was evaluated though rhinoscopy, and samples for histopathology and microbiological cultures were collected. Histopathology revealed chronic infiltration of mature lymphocytes and plasma cells, leading to a diagnosis of chronic lymphoplasmacytic rhinitis. No fungal growth was observed, but bacterial cultures revealed growth of an atypical bacterium mistakenly identified with conventional bacterial identification methods. This isolate was later identified as *P. aestus* by sequencing followed by homologous sequences analysis, corresponding to an environmental strain of *Pseudomonas* used in agricultural settings as a biocide. Antimicrobial susceptibility testing was performed, and this *P. aestus* isolate presented a multidrug resistant profile. *Pseudomonas* are ubiquitous bacteria frequently described as animal and human opportunistic pathogens, and *P. aeruginosa* is the principal species related with animal disease. This work aims to report the first case of animal disease related with *P. aestus*, to the author’s best knowledge. Furthermore, it highlights the need to establishing protocols aiming at the identification and characterization of non-traditional, multidrug-resistant *Pseudomonas* in the clinical setting.

**Abstract:**

The *Pseudomonas* genus includes ubiquitous bacteria frequently described as animal and human opportunistic pathogens. A 9-year-old cat was referred for rhinoscopy at the Veterinary Hospital of the Faculty of Veterinary Medicine, University of Lisbon, Portugal, for an investigation of the chronic respiratory signs. Upon rhinoscopy, nasal and nasopharyngeal discharge were observed, and the nasal turbinates showed signs of inflammation. The nasal biopsies were evaluated by histopathology and mycological and bacterial cultures. The histopathology revealed chronic lymphoplasmacytic inflammation. The mycological culture was negative, but the bacterial culture revealed the growth of a bacterial isolate in the pure culture, identified as *P. aestus* by the sequencing of a 1750 bp PCR amplicon obtained with BCR1 and BCR2 primers, followed by homologous sequences analysis using the NCBI database. The isolate’s susceptibility profile towards 14 antimicrobials was evaluated through the disk diffusion method, being observed that it presented a multidrug resistance profile. The studies available on this environmental *Pseudomonas* strain focused on its potential use for biocide production and application in agricultural settings, and, to the authors’ best knowledge, there are no reports describing its association with infectious diseases in humans or animals, highlighting the importance of establishing protocols aiming at the identification and characterization of non-traditional, multidrug-resistant *Pseudomonas* in the clinical setting.

## 1. Introduction

*Pseudomonas* is a genus of Gram-negative bacilli with a ubiquitous distribution. Due to their potential to adapt to different conditions, the species within this genus are able to colonize terrestrial and aquatic niches, being found in a wide variety of habitats, including aquatic environments and soils [1,2]. Based on phylogenomic and Multi-locus Sequence Analyses (MLSAs), using the genes 16S rDNA, gyrB, rpoB, and rpoD [3], the *Pseudomonas* genus has been divided into 336 validly named species [4], distributed across three lineages, groups, and subgroups. Many of these species live in association with plants, mostly as saprophytes [2], with some animals, such as birds or mammals, acting as reservoirs. This fact is particularly important due to *Pseudomonas* spp.’s involvement in opportunistic infections in humans and animals [2].

In companion animals, *Pseudomonas* species are frequently associated with the development of bacterial infections in multiple locations [5,6]. In dogs and cats, *Pseudomonas* spp. can cause skin and ear infections [7], systemic and urinary infections [8], rhinosinusitis [5], and oral disease [9]. Because of their multiple intrinsic resistance to several antimicrobials, and their capacity to acquire new resistance mechanisms [10] and virulence determinants [1], these bacterial species are challenging opportunistic pathogens to manage. In fact, they have already been associated with Hospital-Acquired Infections (HAIs) in veterinary hospitals [11], and carbapenem-resistant *P. aeruginosa*, a major *Pseudomonas* species, is classified in the World Health Organization (WHO) priority pathogens list for the research and development of new antibiotics as a Critical Priority pathogen [12]. As such, the monitorization and characterization of antimicrobial-resistant *Pseudomonas* strains in veterinary settings are extremely relevant and should focus not only on prevalent species, such as *P. aeruginosa*, but also on less common ones. *P. aestus* was recently described as a new species isolated from Brazilian mangrove sediments, being related to *P. putida* [13]. To the authors’ best knowledge, this report is the first description of a multidrug-resistant *P. aestus* isolate, obtained from the upper airways of a cat with chronic rhinitis. This study aimed to report the association of a non-traditional *Pseudomonas* strain with animal disease and highlight the adversities that can occur in the identification of these bacteria by conventional laboratory procedures.

## 2. Case Presentation

A 9-year-old Chartreux cat was referred to the Veterinary Hospital of the Faculty of Veterinary Medicine of the University of Lisbon, Portugal, for the investigation of the underlying causes of a chronic bilateral nasal discharge.

This cat, an indoor neutered male, was previously submitted to several cycles of antibiotic treatment with doxycycline for a presumed respiratory infection, but the clinical signs persisted. In the first-opinion consultation, a complete blood count and biochemical panel were conducted, in which the only alteration found was a slight hyperglobulinemia (5.2 g/dL; reference level: 2.8–5.1 g/dL).

In the reference hospital, a rhinoscopy with a rigid endoscope (Storz^®^ multi-purpose rigid telescope, Storz, Tuttlingen, Germany) and a transoral nasopharyngoscopy with a flexible endoscope (Storz^®^ broncho-fiberscope) were performed. The main alterations observed included the presence of a mucopurulent bilateral nasal and pharyngeal discharge. The nasal turbinates showed signs compatible with inflammation (congestion, hyperemia, and increased fragility), mainly in the right nasal cavity, but the architecture was maintained. The left nostril and nasopharynx showed no alterations other than the mucopurulent discharge. The right nasal cavity was biopsied via direct visualization and blind nasal biopsies. Samples were collected for histopathological evaluation, and also for mycological and bacterial cultures.

**Figure 1:**
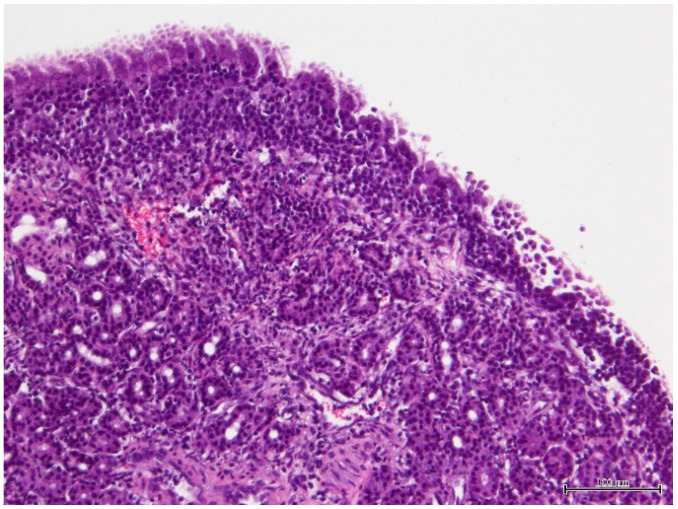
Photomicrograph of the histopathological preparation of the nasal fragment showing chronic lymphoplasmacytic rhinitis. The microscopic evaluation of right nasal cavity biopsy revealed a chronic inflammatory cell infiltrate composed of mature lymphocytes and plasma cells in a moderate amount with slight hyperplasia of submucosal glands (hematoxylin and eosin; bar = 100 µm).

Histopathology analysis revealed an infiltration of the nasal mucosa by mature lymphocytes and plasma cells in a moderate amount, compatible with chronic lymphoplasmacytic rhinitis (Figure 1).

For mycological culture, the sample was inoculated in Sabouraud dextrose agar (VWR, Leuven, Belgium) and incubated at 27 °C for 10 days. After 10 days of incubation, no growth was observed, and the mycological culture was considered negative.

For aerobic bacterial culture, the collected sample was inoculated in Columbia agar + 5% Sheep Blood (COS, bioMérieux, Marcy-l’Etoile, France), MacConkey agar (Oxoid, Hampshire, UK), and Brain Heart Infusion Broth (Oxoid, Hampshire, UK) and incubated at 37 °C for 24 h. After incubation, it was possible to observe the moderate growth of small grayish colonies in COS, and of non-lactose fermenting colonies in MacConkey agar plates, in pure culture. After further isolation in COS at room temperature, the isolate was characterized by Gram staining and oxidase reaction using Bactident^®^ Oxidase (Merck, Darmstadt, Germany), showing oxidase-positive Gram-negative rods, and using the biochemical gallery API 20NE (bioMérieux, Marcy-l’Etoile, France) as *Burkholderia cepacia*.

Since the morphology of the colonies was atypical for this species, a PCR was performed to confirm the identification of the clinical isolate using the primers BCR1 and BCR2 designed to differentiate *Burkholderia* species [14] and total DNA from *B. cenocepacia* K56-2 as a positive control. For that, bacterial broth cultures (clinical isolate and control) were carried out in Lennox broth (containing tryptone 10 g/L, yeast extract 5 g/L, and NaCl 5 g/L). Then, suspensions were incubated with orbital agitation (250 rpm) at 37 °C for *B. cenocepacia* K56-2 or at room temperature for the clinical isolate. Genomic DNA was extracted from exponentially growing broth cultures using the High Pure PCR Template Preparation Kit (Roche, Vienna, Austria), followed by quantification of DNA concentration using an ND-1000 spectrophotometer (NanoDrop Technologies, ThermoFisher Scientific, Waltham, MA, USA).

**Figure 2:**
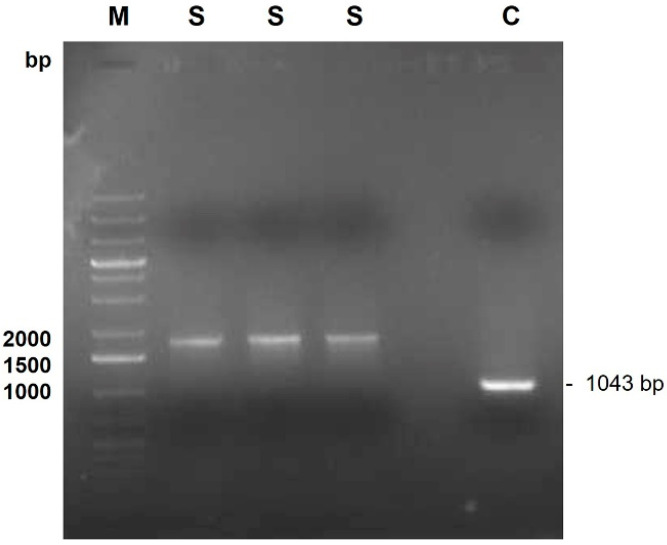
Results from the PCR amplification of the clinical isolate gDNA using the primers BCR1 and BCR2. M—GeneRuler 1 kb Plus DNA Ladder (Thermo Scientific); S—clinical isolate; C—*B. cenocepacia* K56-2 (positive control).

Amplification of the 1043 bp recA gene fragment was performed using the oligonucleotides BCR1 (5′-TGACCGCCGAGAAGAGCAA-3′) and BCR2 (5′-CTCTTCTTCGTCCATCGCCTC-3′) as previously described [14]. Approximately 50 ng of DNA was transferred to a tube with 20 µL of reaction mix containing 1.5 U Taq DNA polymerase (Citomed, Odivelas, Portugal), 250 µM (each) deoxynucleoside triphosphate, 1.5 mM MgSO_4_, 1× PCR buffer (Citomed, Odivelas, Portugal), and 20 pmol of each primer. Amplification was carried out using the 2720 Thermal cycler (Applied Biosystems, ThermoFisher Scientific, Waltham, MA, USA). Samples were initially heated at 95 °C for 3 min before amplification using 30 cycles consisting of 45 s of denaturation at 95 °C, 30 s of annealing at 58 °C, and 63 s of extension at 72 °C. PCR was completed with a final extension step at 72 °C for 7 min. PCR-amplified products were analyzed by electrophoresis in 1% (wt/vol) agarose gels using standard procedures [15]. Molecular size marker Gene Ruler 1 kb Plus DNA Ladder (ThermoFisher Scientific, Waltham, MA, USA) was used. DNA products were stained with GreenSafe Premium (NZYTech, Lisbon, Portugal) and viewed under UV light. After PCR, the amplification of a DNA fragment with around 1043 bp was observed for the control as expected, and 1750 bp for the clinical isolate (Figure 2) (Supplementary Material S1).

The DNA fragments were excised from the agarose gel and purified using NZYGelpure kit (NZYTech, Lisbon, Portugal) according to the manufacturer’s instructions. The purified DNA samples were sequenced by Eurofins Genomics (Ebersberg, Germany) using the BCR1 or BCR2 primers. The BLASTN tool [16] was used for homologous sequences analysis, comparing the obtained sequences from the DNA fragment samples against the nucleotide collection (nr/nt) database available at the NCBI (Supplementary Materials S2 and S3). It was observed that both DNA sequences had higher than 98% identity with *Pseudomonas* sp. CMR5c chromosome. The sequence obtained with the BCR1 or BCR2 primers aligned with the *Pseudomonas* sp. CMR5c chromosome regions 3914375 to 3914798 or with regions 3915726 to 3916081, respectively. *Pseudomonas* sp. CMR5c strain is a phenazine- and biosurfactant-producing fluorescent *Pseudomonas* isolated from red cocoyam (*Xanthosoma sagittifolium*) rhizosphere [17], which was recently reclassified as *P. aestus* [18].

Furthermore, the clinical isolate and the *P. aeruginosa* IST27 control strain were streak-inoculated in the same Lennox agar plate [Lennox broth supplemented with 2% agar (Iberagar, Portugal)], occupying each strain about half of the plate. After incubation for 48 h at room temperature, the plates were visualized under ultraviolet and white light and photographed using the Gel Doc XR+ System (Bio-Rad Laboratories, Hercules, CA, USA). The clinical isolate presented fluorescence under UV light, while, as expected, the *Pseudomonas aeruginosa* IST27 strain did not fluoresce when irradiated with UV light. Moreover, as the *P. aestus* CMR5c was previously described as not being able to grow at 37 °C [17], the test was repeated at 37 °C, with the clinical isolate showing no growth in the Lennox agar plates.

Finally, the susceptibility profile of the isolate was determined using the disk diffusion method according to CLSI [19,20]. Briefly, a bacterial suspension with a 0.5 turbidity in the MacFarland scale was prepared and inoculated over the surface of Mueller–Hinton agar plates (Oxoid, Hampshire, UK), after which antimicrobial disks corresponding to compounds used against *Pseudomonas aeruginosa* (Table 1) were placed onto the agar’s surface. Then, the plates were incubated for 18 h, after which the susceptibility profile of the isolate was determined using the clinical breakpoints for *P. aeruginosa* as a reference. The clinical isolate was susceptible to gentamicin, ceftazidime, piperacillin, and piperacillin-tazobactam, intermediate to tobramycin, amikacin, and to all the quinolones tested, and resistant to imipenem, meropenem, cefepime, and aztreonam. This way, it can be classified as a multidrug-resistant (MDR) isolate according to Magiorakos et al. [21], which considers that both the resistant and intermediate results obtained after the in vitro testing correspond to non-susceptibility (Table 1).

After the procedure, the follow-up of the animal was performed by the first-opinion veterinarian, and no information is available on the infection’s treatment and development.

## 3. Discussion

Feline chronic rhinitis is a frequent condition that can be challenging to manage [22]. In cats, it is characterized by the inflammation of the nasal cavity, which can last for 4 weeks or longer, being the second most frequent cause of chronic nasal discharge in cats after neoplasia [22,23]. The chronic rhinitis signs in cats can have multiple origins, with the primary conditions occasionally involving viral or fungal infections, nasal parasites, neoplasia, congenital defects, dental disease, nasal foreign bodies, nasopharyngeal polyps, allergic rhinitis, and nasal trauma [23]. Nasal cavity abnormalities can be detected through rhinoscopy, as in the present case, but conventional and contrast radiography, magnetic resonance imaging (MRI), and computed tomography (CT) scans are also useful for the full clarification of some situations [24].

In this study, the cat’s nasal cavity presented a moderate lymphoplasmacytic infiltration, which can probably be considered as the primary cause of the nasal discharge observed, and the *P. aestus* presence is likely to be secondary to the underlying cause. Nevertheless, it is known that the presence of chronic inflammation leads to damage of the nasal mucosa and may predispose to secondary bacterial infections [25], which can be difficult to control if promoted by multidrug-resistant strains.

Cat rhinitis cases associated with primary bacterial infections are considered rare. The association of the infection with bacteria is usually promoted by opportunistic pathogens or by an overgrowth of the nasal microbiota and is typically secondary to the primary etiology, such as primary viral infection, especially by feline herpesvirus 1 (FHV-1) [22]. Although the mixed growth of commensal microorganisms is frequently observed, the single presence of a pathogenic species, including *P. aeruginosa*, can be more significant [25]. In these cases, cats with chronic lymphoplasmacytic rhinitis can benefit from antibacterial therapy [24].

*Pseudomonas* spp. are common organisms identified when deeper nasal samples are obtained from cats with chronic rhinosinusitis [25]. Although this bacterial genus includes ubiquitous bacteria that are frequently described as animal and human opportunistic pathogens, to the authors’ best knowledge, this is the first report of the presence of *P. aestus* isolate associated with disease in a mammal. To date, the few studies available on this environmental *Pseudomonas* strain focused on its potential use as a biocontrol agent in the food industry and agriculture. In fact, it presents broad-spectrum antimicrobial activity associated with the ability to produce pyrrolnitrin, pyoluteorin, 2,4-diacetylphloroglucinol, hydrogen cyanide, and exoprotease [17]. The nasal inflammation present in the animal may have allowed the invasion of its nasal cavity by this environmental agent.

Since this can be a chronic and recurrent situation, the administration of antimicrobial therapy for 6 to 8 weeks may be necessary [22]. If so, it should ideally rely on the susceptibility profile of the isolates obtained from the nasal biopsies or nasal flush fluid. The presence of bacteria in biopsy samples suggests a more established infection than the colonization of the epithelial surface [25]. Since cats with chronic rhinitis often have a transient response to antimicrobial treatment, the recurrence of the disease is frequent, suggesting that the bacteria involvement is secondary to the pathogenesis of the disease [25], and repeated antibacterial courses may be required, contributing to the elimination of other commensal organisms and the selection of resistant strains. In fact, some authors highlight that repeated courses of antibiotics may result in selection for *Pseudomonas* spp. [22]. In this case, the fact that the animal was previously subjected to several cycles of doxycycline treatment can be one possible explanation for the selection of this bacterial species and the susceptibility pattern presented by this clinical isolate, which may make the control and elimination of the associated infection more difficult. 

A limitation of this study is the absence of the follow-up clinical information of the animal; however, the main goal of this work was to report a non-conventional bacterial species responsible for a nasal infection in a cat.

Concluding, this manuscript constitutes the first report of the isolation of a multidrug-resistant, non-traditional *P. aestus* strain in companion animal medicine, highlighting the possibility that less common *Pseudomonas* species, including environmental strains, can cause disease in animals and remain unnoticed due to the difficulties associated with their identification using the conventional bacteriological methods. The resistance profile of the clinical isolate under study also supports the establishment of monitorization protocols aiming at the isolation, identification, and characterization of non-traditional *Pseudomonas* strains in the veterinary setting.

## Figures and Tables

**Table 1:** Antimicrobial resistance profile of the *Pseudomonas aestus* clinical isolate under study.

Antimicrobial Class	Antimicrobial Agent	Disk Content	Inhibition Halo Diameter (mm)	Classification
Aminoglycosides	Gentamicin	10 µg	17	Susceptible
	Tobramycin	10 µg	14	Intermediate
	Amikacin	30 µg	16	Intermediate
Carbapenems	Imipenem	10 µg	8	Resistant
	Meropenem	10 µg	6	Resistant
Cephalosporins	Ceftazidime	30 µg	21	Susceptible
	Cefepime	30 µg	6	Resistant
Antipseudomonal penicillin	Piperacillin	100 µg	22	Susceptible
Antipseudomonal penicillin + β-lactamase inhibitor	Piperacillin/tazobactam	100/10 µg	21	Susceptible
Monobactam	Aztreonam	30 µg	6	Resistant
Fluoroquinolones	Ciprofloxacin	5 µg	20	Intermediate
	Enrofloxacin	5 µg	19	Intermediate
	Marbofloxacin	5 µg	18	Intermediate
	Ofloxacin	5 µg	14	Intermediate

## Data Availability

The datasets used and/or analyzed during the current study are available from the corresponding author upon reasonable request.

## Supplementary Materials

The following supporting information can be downloaded at https://www.mdpi.com/article/10.3390/vetsci11080382/s1, Supplementary Material S1: original electrophoresis gel image; Supplementary Material S2: Sequences of the fragments amplified using BCR1 and BRC2 primers; Supplementary Material S3: Sequence alignment results with BCR1 and BCR2 primers using the BLASTN tool.
