# Supplementary material for: Pseudomonas aestus Isolation from the Nasal Cavity of a Cat with Chronic Rhinitis
Source: Vet Sci. 2024 Aug 19;11(8):382. doi: 10.3390/vetsci11080382 (PMC11359275; doi:10.3390/vetsci11080382)
Supplement: Supplementary file 1 [file vetsci-11-00382-s001.zip › Supplementary Material_2.pdf]

Supplementary material 1

1.1. Sequence BCR1. Sequence of the fragment amplified using BCR1 primer

>FMV_BCR1_Premixed (428 nt)

CAACGGACAGGTCGCGGCCCTGGGCCAACGAAAGCGCCGCACTACACGACACCCGCA

ACATGCTGCAGGGCGAACGGACCCACAATGCCGAACTCGGCCAGGCCCTCAAGGACTA

CGACCTGCGCCTGGCCGACAAGGAACAGCAGATCCGCTCCCTGGAAGAAAAGCACCTG

CATGCCCGCGACGCCCTGGAGCATTACCGCGCAGCCAGCAAGGAGCAGCGCGACCAG

GAACTGCGTCGCCACGAGGGCCAAATGCAGCAAGTCCAGATGGAGTTGCGCCAGGCCC

AGCAGAGCCTCCTGGTACGCCAGGATGAGATCACCCAGCTGCACCGCGACAACGAACG

CCTGCTCAGCGAGCAACGCACCACTCTCAAGGAACTCAAACAGACCCAGGAAGCGCTG

AGCCAGAGCAGCACTCTGCAAACG

1.2. Sequence BCR2. Sequence of the fragment amplified using BCR2 primer

>FMV_BCR2_Premixed (362 nt)

ATCGACTTGCTGGTCAACAGCGGGCGTGACCCTGCACGAAGGGCATGCCAAGTTGATC

GATCCGCATCAGGTGGAGGTCAATGGCCAGCGCTATACCGCCAGGCACATCCTGATCG

CCACTGGCGGCTGGCCGCAGATCCCGGACATCCCGGGGCGCGAACATGCCATCAGCTC

CAATGAAGCCTTCTATCTAAAGGAACTGCCCAAGCGCATCCTGGTGGTGGGCGGCGGC

TACATCGCGGTGGAGTTCGCCGGAATCTTCCACGGCCTGGGGGCGGACACCCAGTTGTT

GTATCGCGGTGACTTGTTCCTGCGCGGCTTCAATGGCGCGGTGCGCAAGCACCTGCAGG

AATAGGTGACCG
