# Supplementary material for: Pseudomonas aestus Isolation from the Nasal Cavity of a Cat with Chronic Rhinitis
Source: Vet Sci. 2024 Aug 19;11(8):382. doi: 10.3390/vetsci11080382 (PMC11359275; doi:10.3390/vetsci11080382)
Supplement: Supplementary file 1 [file vetsci-11-00382-s001.zip › Supplementary Material_3.pdf]

Supplementary material 2

2.1. Sequence alignment results with BCR1 primer, using the BLASTN tool

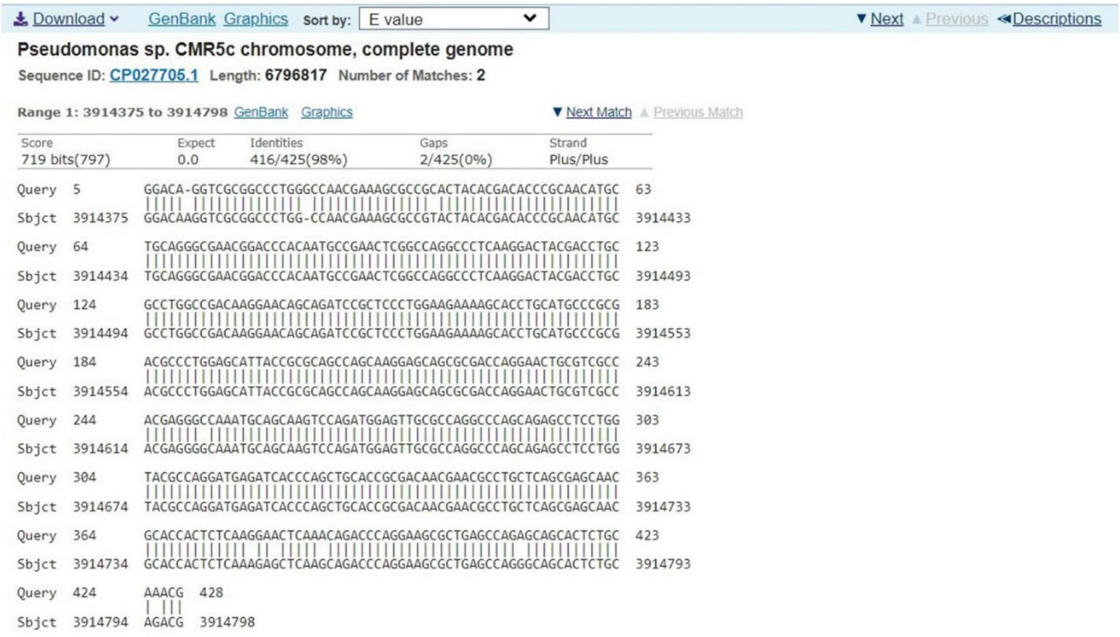

Download GenBank Graphics Sort by: E value ▼ Next ▲ Previous ◀◀ Descriptions

Pseudomonas sp. CMR5c chromosome, complete genome

Sequence ID: CP027705.1 Length: 6796817 Number of Matches: 2

Range 1: 3914375 to 3914798 GenBank Graphics ▼ Next Match ▲ Previous Match

Score	Expect	Identities	Gaps	Strand
719 bits(797)	0.0	416/425(98%)	2/425(0%)	Plus/Plus

```
Query  5        GGACA-GGTCGCGGCCCTGGGCCAACGAAAGCGCCGCACTACACGACACCCGCAACATGC  63
                ||||| |||||||||||||| ||||||||||||||| |||||||||||||||||||||||
Sbjct  3914375  GGACAAGGTCGCGGCCCTGG-CCAACGAAAGCGCCGTACTACACGACACCCGCAACATGC  3914433

Query  64       TGCAGGGCGAACGGACCCACAATGCCGAACTCGGCCAGGCCCTCAAGGACTACGACCTGC  123
                ||||||||||||||||||||||||||||||||||||||||||||||||||||||||||||
Sbjct  3914434  TGCAGGGCGAACGGACCCACAATGCCGAACTCGGCCAGGCCCTCAAGGACTACGACCTGC  3914493

Query  124      GCCTGGCCGACAAGGAACAGCAGATCCGCTCCCTGGAAGAAAAGCACCTGCATGCCCGCG  183
                ||||||||||||||||||||||||||||||||||||||||||||||||||||||||||||
Sbjct  3914494  GCCTGGCCGACAAGGAACAGCAGATCCGCTCCCTGGAAGAAAAGCACCTGCATGCCCGCG  3914553

Query  184      ACGCCCTGGAGCATTACCGCGCAGCCAGCAAGGAGCAGCGCGACCAGGAACTGCGTCGCC  243
                ||||||||||||||||||||||||||||||||||||||||||||||||||||||||||||
Sbjct  3914554  ACGCCCTGGAGCATTACCGCGCAGCCAGCAAGGAGCAGCGCGACCAGGAACTGCGTCGCC  3914613

Query  244      ACGAGGGCCAAATGCAGCAAGTCCAGATGGAGTTGCGCCAGGCCCAGCAGAGCCTCCTGG  303
                ||||||| ||||||||||||||||||||||||||||||||||||||||||||||||||||
Sbjct  3914614  ACGAGGGGCAAATGCAGCAAGTCCAGATGGAGTTGCGCCAGGCCCAGCAGAGCCTCCTGG  3914673

Query  304      TACGCCAGGATGAGATCACCCAGCTGCACCGCGACAACGAACGCCTGCTCAGCGAGCAAC  363
                ||||||||||||||||||||||||||||||||||||||||||||||||||||||||||||
Sbjct  3914674  TACGCCAGGATGAGATCACCCAGCTGCACCGCGACAACGAACGCCTGCTCAGCGAGCAAC  3914733

Query  364      GCACCACTCTCAAGGAACTCAAACAGACCCAGGAAGCGCTGAGCCAGAGCAGCACTCTGC  423
                ||||||||||||| || ||||| |||||||||||||||||||||||| ||||||||||||
Sbjct  3914734  GCACCACTCTCAAAGAGCTCAAGCAGACCCAGGAAGCGCTGAGCCAGGGCAGCACTCTGC  3914793

Query  424      AAACG  428
                | |||
Sbjct  3914794  AGACG  3914798
```

2.2. Sequence alignment results with BCR2 primer, using the BLASTN tool

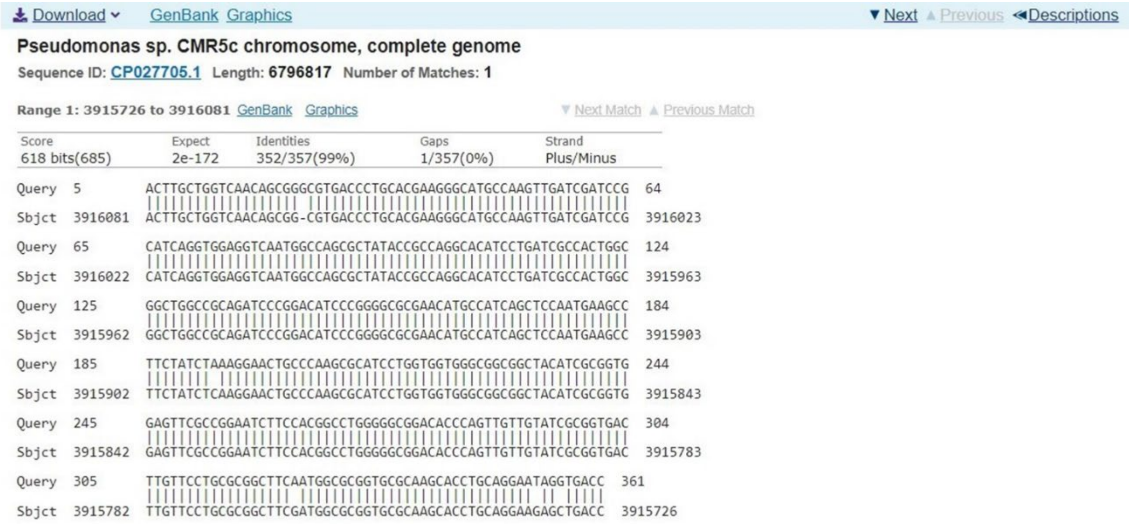

Download GenBank Graphics ▼ Next ▲ Previous ◀◀ Descriptions

Pseudomonas sp. CMR5c chromosome, complete genome

Sequence ID: CP027705.1 Length: 6796817 Number of Matches: 1

Range 1: 3915726 to 3916081 GenBank Graphics ▼ Next Match ▲ Previous Match

Score	Expect	Identities	Gaps	Strand
618 bits(685)	2e-172	352/357(99%)	1/357(0%)	Plus/Minus

```
Query  5        ACTTGCTGGTCAACAGCGGGCGTGACCCTGCACGAAGGGCATGCCAAGTTGATCGATCCG  64
                ||||||||||||||||||| ||||||||||||||||||||||||||||||||||||||||
Sbjct  3916081  ACTTGCTGGTCAACAGCGG-CGTGACCCTGCACGAAGGGCATGCCAAGTTGATCGATCCG  3916023

Query  65       CATCAGGTGGAGGTCAATGGCCAGCGCTATACCGCCAGGCACATCCTGATCGCCACTGGC  124
                ||||||||||||||||||||||||||||||||||||||||||||||||||||||||||||
Sbjct  3916022  CATCAGGTGGAGGTCAATGGCCAGCGCTATACCGCCAGGCACATCCTGATCGCCACTGGC  3915963

Query  125      GGCTGGCCGCAGATCCCGGACATCCCGGGGCGCGAACATGCCATCAGCTCCAATGAAGCC  184
                ||||||||||||||||||||||||||||||||||||||||||||||||||||||||||||
Sbjct  3915962  GGCTGGCCGCAGATCCCGGACATCCCGGGGCGCGAACATGCCATCAGCTCCAATGAAGCC  3915903

Query  185      TTCTATCTAAAGGAACTGCCCAAGCGCATCCTGGTGGTGGGCGGCGGCTACATCGCGGTG  244
                |||||||| |||||||||||||||||||||||||||||||||||||||||||||||||||
Sbjct  3915902  TTCTATCTCAAGGAACTGCCCAAGCGCATCCTGGTGGTGGGCGGCGGCTACATCGCGGTG  3915843

Query  245      GAGTTCGCCGGAATCTTCCACGGCCTGGGGGCGGACACCCAGTTGTTGTATCGCGGTGAC  304
                ||||||||||||||||||||||||||||||||||||||||||||||||||||||||||||
Sbjct  3915842  GAGTTCGCCGGAATCTTCCACGGCCTGGGGGCGGACACCCAGTTGTTGTATCGCGGTGAC  3915783

Query  305      TTGTTCCTGCGCGGCTTCAATGGCGCGGTGCGCAAGCACCTGCAGGAATAGGTGACC  361
                |||||||||||||||||| ||||||||||||||||||||||||||||| || |||||
Sbjct  3915782  TTGTTCCTGCGCGGCTTCGATGGCGCGGTGCGCAAGCACCTGCAGGAAGAGCTGACC  3915726
```
