# Supplementary figures and images for: Pseudomonas aestus Isolation from the Nasal Cavity of a Cat with Chronic Rhinitis
Source: Vet Sci. 2024 Aug 19;11(8):382. doi: 10.3390/vetsci11080382 (PMC11359275; doi:10.3390/vetsci11080382)

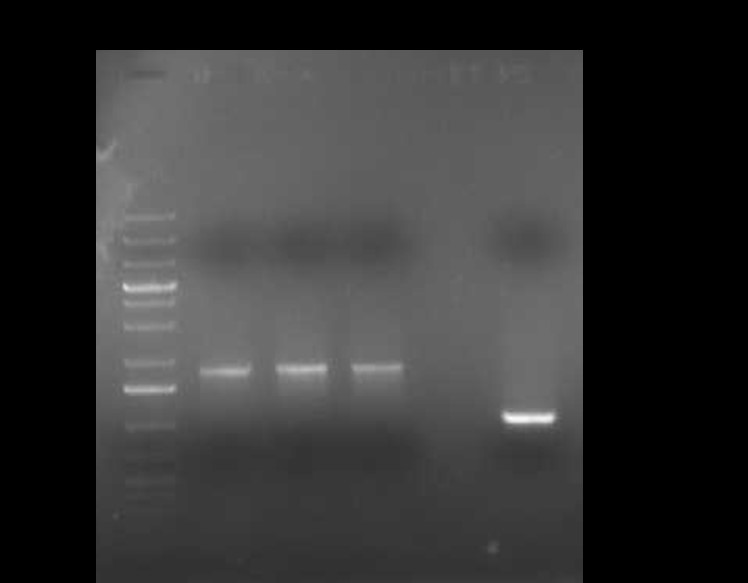

Supplement: Supplementary file 1 [file vetsci-11-00382-s001.zip › Supplementary Material_1.TIFF]
